# Necrotizing Enterocolitis (NEC) and the Risk of Intestinal Stricture: The Value of C-Reactive Protein

**DOI:** 10.1371/journal.pone.0076858

**Published:** 2013-10-11

**Authors:** Aurélie Gaudin, Caroline Farnoux, Arnaud Bonnard, Marianne Alison, Laure Maury, Valérie Biran, Olivier Baud

**Affiliations:** 1 Neonatal Intensive Care Unit, Robert Debré Children University Hospital and Denis Diderot Paris University, APHP, Paris, France; 2 Department of General Pediatric Surgery, Robert Debré Children University Hospital and Denis Diderot Paris University, APHP, Paris, France; 3 Department of Pediatric Radiology, Robert Debré Children University Hospital and Denis Diderot Paris University, APHP, Paris, France; New York University, United States of America

## Abstract

Necrotizing enterocolitis (NEC) is a severe complication frequently seen during the neonatal period associated with high mortality rate and severe and prolonged morbidity including Post-NEC intestinal stricture. The aim of this study is to define the incidence and risk factors of these post-NEC strictures, in order to better orient their medicosurgical care. Sixty cases of NEC were retrospectively reviewed from a single tertiary center with identical treatment protocols throughout the period under study, including systematic X-ray contrast study. This study reports a high rate of post-NEC intestinal stricture (n = 27/48; 57% of survivors), either in cases treated surgically (91%) and after the medical treatment of NEC (47%). A colonic localization of the strictures was more frequent in medically-treated patients than in those with NEC treated surgically (87% vs. 50%). The length of the strictures was significantly shorter in case of NEC treated medically. No deaths were attributable to the presence of post-NEC stricture. The mean hospitalization time in NICU and the median age at discontinuation of parenteral nutrition were longer in the group with stricture, but this difference was not significant. The median age at discharge was significantly higher in the group with stricture (p = 0.02). The occurrence of post-NEC stricture was significantly associated with the presence of parietal signs of inflammation and thrombopenia (<100 000 platelets/mm^3^). The mean maximum CRP concentration during acute phase was significantly higher in infants who developed stricture (p<0.001), as was the mean duration of the elevation of CRP levels (p<0.001). The negative predictive value of CRP levels continually <10 mg/dL for the appearance of stricture was 100% in our study. In conclusion, this retrospective and monocentric study demonstrates the correlation between the intensity of the inflammatory syndrome and the risk of secondary intestinal stricture, when systematic contrast study is performed following NEC.

## Introduction

Necrotizing enterocolitis (NEC) is a severe complication frequently seen during the neonatal period. In the USA, almost one newborn in 1000 is hospitalized each year for NEC [1], [2] and 2–7% of newborns hospitalized in neonatal intensive care units are affected by the disease [1], [3]–[7]. This is a serious illness with an estimated mortality rate of 15–35% [1], [8]. Despite a significant decrease in the mortality of very premature infants, the mortality rate of NEC cases has increased [3].

Post-NEC intestinal stricture has been described in 1968 by Rabinowitz [9], and 1970 by Krasna [10]. One of the first reviews in the literature, published in 1980, mentions 29 cases [11]. Intestinal stricture at the time was estimated to affect 20% of NEC survivors [11]–[13] and was associated with severe and prolonged morbidity (septicemia, perforation, intestinal obstruction) [14]–[16]. These data led many authors to recommend systematic contrast study of the digestive tract [11], [17], [18]. Since then, the reported incidence of detectable stricture has increased (to up to 40% of NEC cases), and is higher after initial surgical treatment (20–43%) than after medical treatment (15–30%) [17], [19]–[22].

The treatment of post-NEC strictures, in particular when they are asymptomatic, is controversial (modalities of screening, indications for surgery, optimal time). Moreover, no validated risk factor was reported to better select neonates, at risk of intestinal stricture, who could benefit from contrast study of the digestive tract.

This study describes a homogenous population of NEC cases with secondary stricture, diagnosed and treated in a pluridisciplinary pediatric center. Our aim is to define the incidence and risk factors of these post-NEC strictures, in order to better orient their medicosurgical care.

## Patients and Methods

### Population Studied

This is a retrospective study that includes all neonates born between 1 January 1999 and 31 December 2006 and hospitalized in the neonatal intensive care unit of Robert Debre Hospital, with a diagnosis of NEC (≥ Bell’s stage II, modified by Walsh [23]) confirmed during hospitalization, and for whom the entire course of treatment took place at this center. NEC cases referred from other perinatal centers for the treatment of intestinal stricture were thus excluded. Retrospective review of our data collection was approved by our institutional review board (IRB) and local ethical committee named “Comité de l’Evaluation de l’Ethique des Projets de Recherche Biomédicale de Robert Debré (CEERB-RD)”. An information letter was sent to parents for this retrospective study but no written consent has been required by IRB.

Since the difference between suspected and confirmed NEC lies in the presence or absence of pneumatosis, all radiological results were read by two independent investigators in order to confirm the NEC stage. In all cases, these were obtained by standard X-ray examination. Examinations were repeated every 6 hours during acute phase of suspected NEC. C-reactive protein (CRP) levels were measured in all infants in the hours following the onset of NEC. This test was repeated at least once every 24 h for the first 48 h.

### Treatment Protocols

The treatment protocols for NEC were identical throughout the period under study. The first-line treatment was medical, combining the cessation of feeding with gentle suction (−10 cm H_2_0), broad-spectrum antibiotics, the maintenance of vital hemodynamic and respiratory function, analgesia and parenteral nutrition.

During the acute phase, the only absolute indication for immediate surgery was pneumoperitoneum. A relative indication was extended digestive necrosis with inflammation or sepsis not controlled by medical treatment. In all operated infants, the surgical protocol used was explorative laparotomy, an examination of the entire digestive tract and the search for perforations, sampling for bacteriology and the creation of a proximal stoma for evacuation. In all cases, contrast study was carried out.

### Statistical Analysis

Results were analyzed in two steps from our database: a descriptive analysis of the whole population and of the group with stricture, and a comparative analysis of groups with and without stricture.

Descriptive data are expressed (for quantitative variables) as means ± standard deviations for homogeneous distributions, and by the median and interquartile range (IR) for heterogeneous distribution; for qualitative variables, data are expressed as percentages.

Comparative analyses are univariate, using the Chi^2^ test for qualitative variables, with the Yates correction if necessary, and the Student’s t test for quantitative variables. The threshold for significance was fixed at p = 0.05.

## Results

### Characteristics, Treatment and Prognosis of NEC

We studied 60 cases of NEC that corresponded to the inclusion criteria. Their distribution according to Bell’s classification, as modified by Walsh, is presented in Table 1 and their clinical and biological description in Table 2.

**Table 1:** Staging and distribution of NEC (according to Bell, modified by Walsh).

	%	(n)
Grade 2a	46	(28)
Grade 2b	12	(7)
Grade 3a	20	(12)
Grade 3b	22	(13)

**Table 2:** Clinical, biological and radiological description of NEC.

	n (%)
Rectorrhagia	38 (63)
Respiratory and hemodynamic instability	27 (45)
Metabolic acidosis	15 (25)
Disseminated intravascular coagulopathy	12 (20)
Thrombopenia*	15 (29)
Neutropenia*	20 (38)
CRP always <10 mg/L*	8 (15)
Portal venous gas	20 (33)
Pneumoperitoneum	6 (10)
Pathogenic agent	18 (30)
Positive blood culture n = 12	
Virology of stools (Adeno/Rotavirus) n = 3	

* 8 cases of fulminant NEC for which data are not available were excluded.

Twenty-nine infants (49%) required assisted ventilation and/or hemodynamic support by fluid expansion and/or the administration of vasoactive amines. It should be noted that only 8 infants (i.e. 15%) did not display an inflammatory syndrome as defined by a CRP value of >10 mg/l, after the exclusion of 8 cases of fulminant NEC in which biological samples could be obtained only once (due to the death of the infant within a few hours after the beginning of symptoms).

Fourteen infants (23%) needed early surgical treatment during the acute phase: 11 for pneumoperitoneum and 3 for the failure of medical treatment and uncontrolled sepsis. The surgical intervention took place after a median time period of 3 days (IR: 1–13) after the beginning of symptoms. A proximal stoma was created in all cases except one, in which almost the entire digestive tract was necrotic and no further action could be taken.

Fourteen infants (23% of NEC cases) died, including one who died at one year with severe bronchopulmonary dysplasia. The other deaths directly attributed to NEC were (i) 12 deaths during the acute phase (on average on day 5±7 following the beginning of symptoms), yielding a mortality rate of 20% during the acute phase; among these, 8 infants, i.e. 13% of NEC cases, presented fulminant NEC with the establishment of severe multiorgan failure within a few hours, and (ii) one death on day 34, related to cystic periventricular leukomalacia.

### Characteristics of Post-NEC Strictures in Surgically-treated Patients

Ten of the 14 patients treated surgically survived the acute phase of NEC. Feeding was reestablished after a median waiting time of 28 days (IR: 24–38). In all cases, contrast study was carried out after a mean waiting period of 48 days (±11) from stoma creation (median 45 days; IR: 43–55). In 6 cases, contrast study led to the diagnosis of one or more strictures, which were surgically resected at the time of reestablishment of continuity. In 3 infants, contrast study did not reveal detectable strictures: however, in one case, an examination of the digestive tract at the time of reestablishment of continuity permitted the diagnosis of staged ileal strictures; in the 2 other cases, the diagnosis only occurred secondary to the appearance of an intestinal obstruction after the reestablishment of continuity.

**Figure 1:**
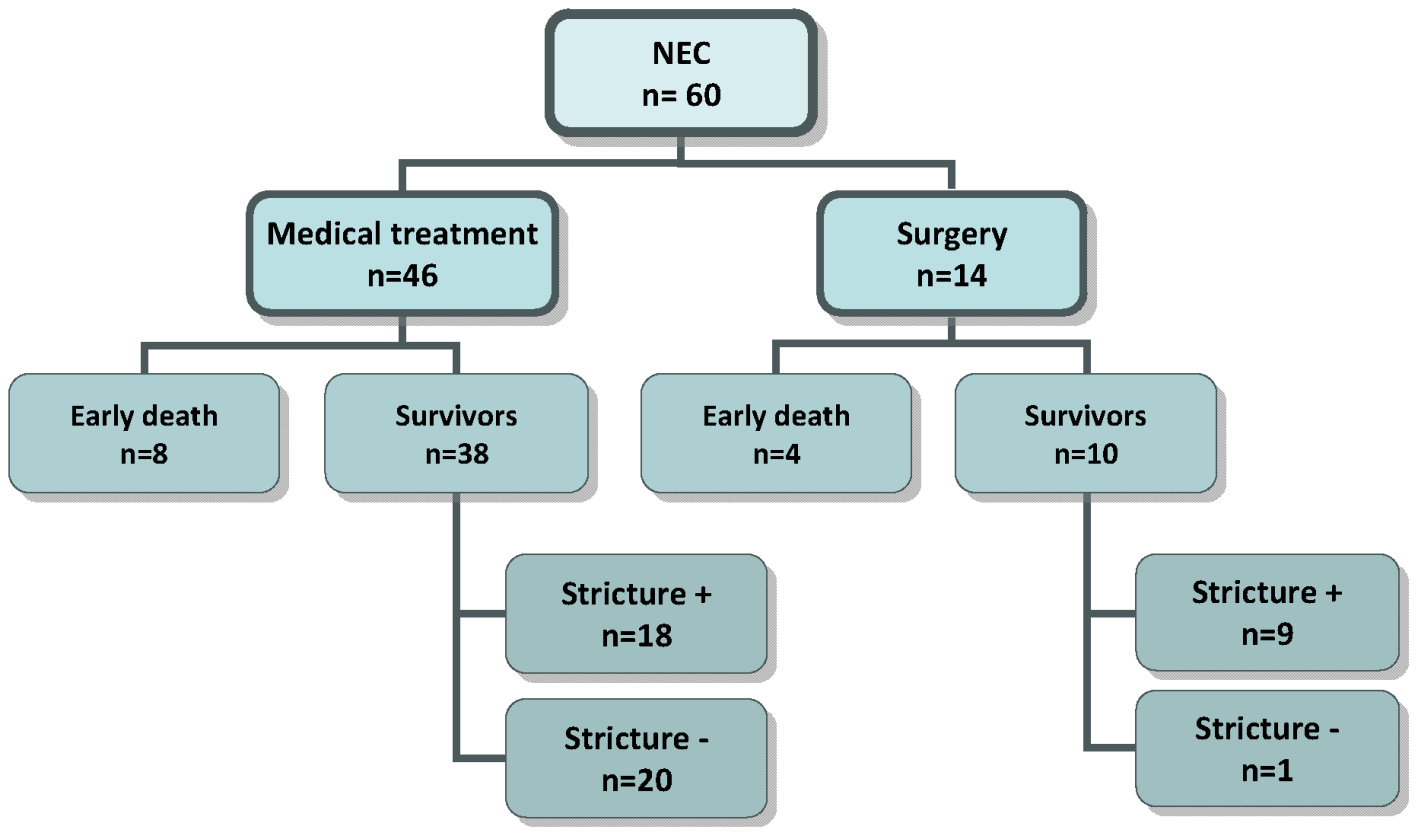
Evolution of NEC in the studied population.

A single infant displayed no intestinal stricture, bringing the frequency of stricture to 90% among survivors, and 64.2% among the entire NEC population operated on during the acute phase (Figure 1). In all cases except one, the strictures could be resected during a single surgical session with the reestablishment of continuity, after a mean waiting period of 63 days following NEC. The macroscopic characteristics of strictures secondary to surgically-treated NEC are summarized in Table 3.

**Table 3:** Macroscopic characteristics of strictures.

	Medically-treatedNEC	Surgically-treated NEC
	18 patients – 30 strictures	10 patients – 16 strictures
Localization	Colon: 26 (87%)	Terminal ileum: 8 (50%)
**Number of strictures:**		
1	17 (56%)	11 (70%)
2	8 (27%)	2 (10%)
3	5 (17%)	3 (20%)
**Length of strictures** * **:**		
Short <2 cm	25 (83%)	9 (56%)
Long ≥5 cm	1 (3%)	7 (44%)

* statistically significant differences between the two groups of NEC (p<0.01).

### Characteristics of Post-NEC Strictures in Medically-treated Patients

Thirty-eight of the 46 infants treated medically survived the acute phase of NEC, with a median treatment duration from the cessation of feeding of 21 days (IR: 16–24). In 34 infants, digestive contrast study was performed after a median waiting time of 22 days (IR: 18–24) after the beginning of NEC. In 65% of cases (n = 22), it was carried out before the reestablishment of feeding. The contrast study revealed one or more intestinal strictures in 18 cases (53%).

The frequency of intestinal stricture was thus 39.5% in the entire population of NEC cases treated medically, and 47.3% among survivors.

**Figure 2:**
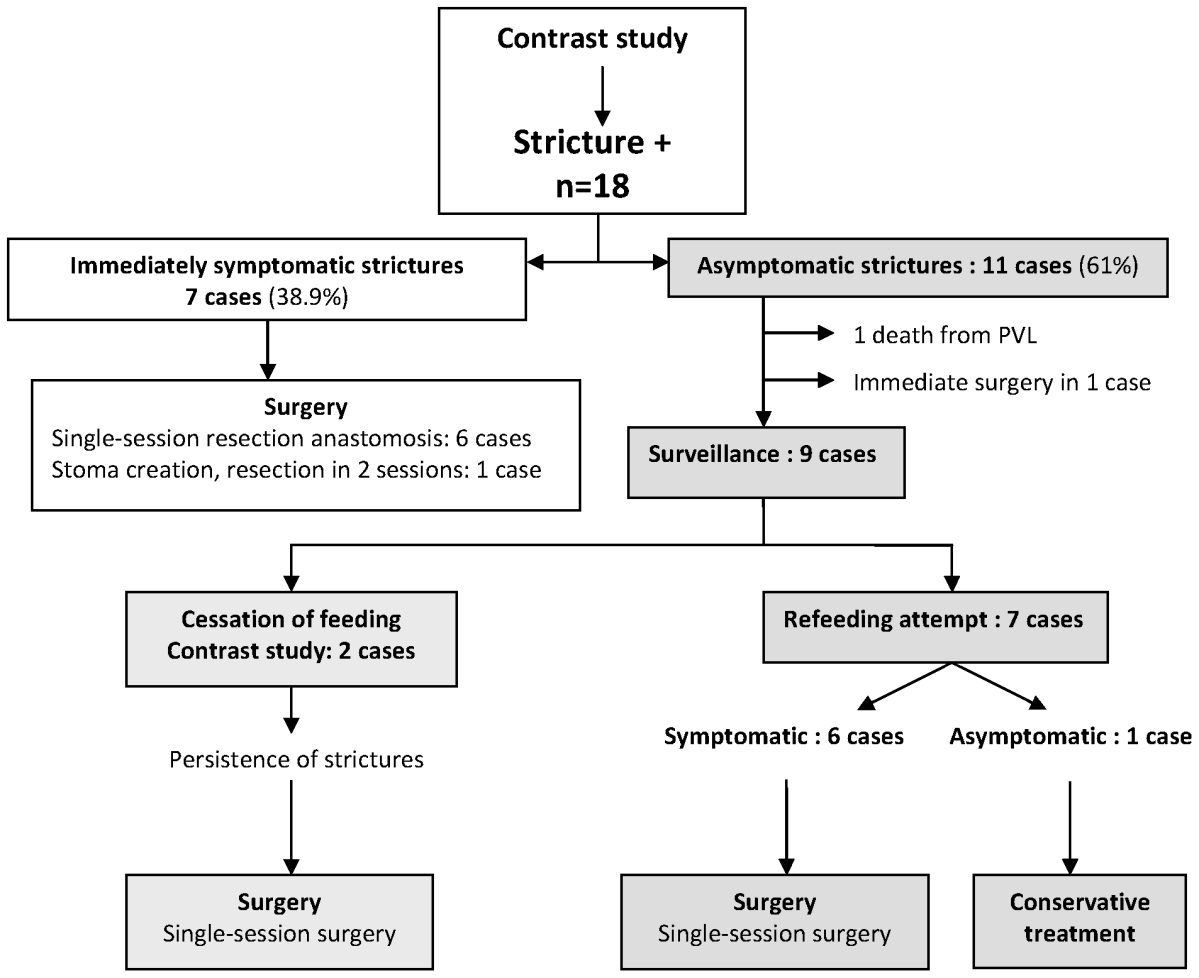
Treatment of strictures after contrast study (NEC cases treated medically during the acute phase).

The treatment followed after diagnosis in the 18 cases with stricture is summarized in Figure 2. A single case of stricture was successfully treated medically, despite the persistence of stricture at 6 weeks following NEC. In 16 cases, surgical treatment was necessary, either immediately (7 symptomatic and 3 asymptomatic infants) or after the failure of refeeding (6 cases). The intervention took place after a median waiting time of 36 days (IR: 32–46) following NEC. Fifteen of the 16 cases underwent single-session surgery for resection and anastomosis.

The strictures were symptomatic in 13/18 cases, after a time period of 23 days (IR: 21–26) following NEC and 2 days (IR: 0–7) following the reestablishment of feeding. In 3 cases, it presented as septicemia of digestive origin, in 6 cases as an intestinal obstruction-like syndrome and in 4 cases as an obvious intestinal obstruction.

The macroscopic characteristics of stricture secondary to NEC treated medically are summarized in Table 3. A colonic localization of the strictures was more frequent in these patients than in those with NEC treated surgically (87% vs. 50%). The length of the strictures was significantly shorter in case of NEC treated medically (p<0.01; Table 3).

### Complications Associated with Post-NEC Strictures

No deaths were attributable to the presence of post-NEC stricture. In the group of patients with intestinal stricture, the median age at discontinuation of parenteral nutrition was 90 days, and the corrected median age at discharge was 45 weeks (IR: 42–55 weeks; Table 4).

**Table 4:** Comparative table of groups with and without stricture.

	Stricture +(n = 27)	Stricture −(n = 21)	p-value
Gestational age at birth, mean (weeks)	31+6	31+2	NS
Mean weight at birth, g	1693	1479	NS
IUGR, n (%)	3 (11)	5 (24)	NS
Sex male, n (%)	13 (48)	8 (38)	NS
Postmenstrual age at NEC, mean (weeks)	35	35+3	NS
Mean age, days)	21	29	NS
Parietal signs, n (%)	9 (33)	1 (5)	p = 0.039*
Portalvenous gas, n (%)	9 (33)	3 (14)	NS
State of shock, n (%)	6 (22)	2 (10)	NS
Disseminated intravascular coagulopathy, n (%)	3 (11)	1 (5)	NS
Thrombopenia <100 000/mm^3^, n (%)	11 (41)	2 (10)	p = 0.021*
Surgical treatment in acute phase, n (%)	9 (33)	1 (5)	p = 0.039*
Duration of intensive care in days, n (%)	14 (52)	3 (14)	p = 0.016*
Duration of parenteral nutrition after NEC			
≥45 days – n	22	3	
≥90 days – n	8	2	
≥120 days – n	7	0	
≥365 days – n	3	0	
Median age at discontinuation of PN (days)	90	56	p = 0.06
Extended digestive resection, n (%)	6 (21)	0	–
Median age at discharge, days	95	83	p = 0.02*
Postmenstrual age at discharge, mean (weeks)	50+4	43+1	p = 0.009*
Mean weight at discharge, g	4140	3032	p = 0.004*

After the resection and anastomosis of the stricture, 2 infants from surgical group developed at least one secondary stricture, leading to the extended resection of the colon and small intestine.

**Figure 3:**
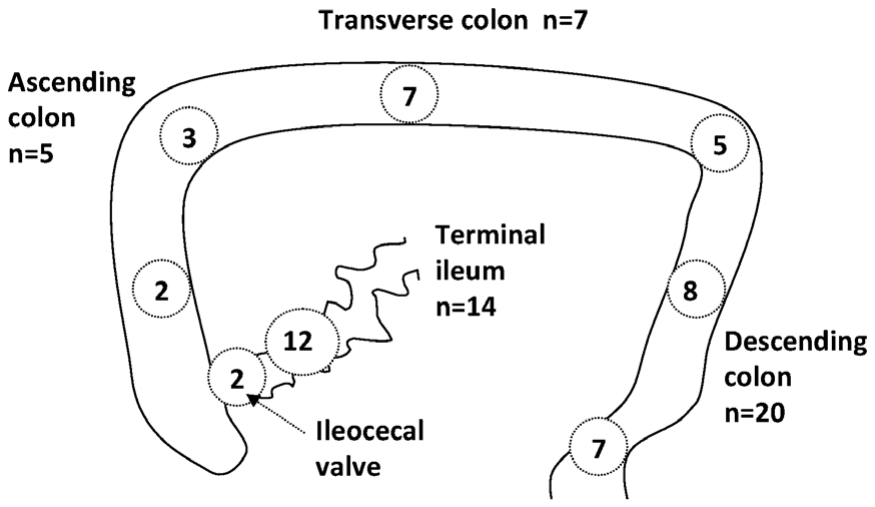
Localization of post-NEC strictures.

Localization of strictures following NEC is depicted in Figure 3. In all, 8 of the 28 patients with a post-NEC intestinal stricture underwent extended digestive resection. In 2 of these patients, this involved extended colonic resection, with no digestive symptoms noted at later time points.

The mean hospitalization time in NICU and the median age at discontinuation of parenteral nutrition were longer in the group with stricture, but this difference was not significant. On the other hand, the median age at discharge was significantly higher in the group with stricture (p = 0.02; Table 4).

### Risk and Predictive Factors for Post-NEC Stricture

The results of the comparative analysis of groups with and without stricture are presented in Table 4. The two groups were not significantly different with respect to sex, gestational age or weight at birth, or with respect to term-corrected age or weight at diagnosis. The occurrence of post-NEC stricture was significantly associated with the presence of parietal signs (erythema, abdominal wall thickening or abdominal guarding) and thrombopenia (<100000 platelets/mm^3^).

C-reactive protein (CRP) levels were measured in all infants in the hours following diagnosis. This test was repeated at least once every 24 h for the first 48 h and its predictive value for the occurrence of post-NEC stricture. In 8 infants, no increase in CRP levels was seen; none of these infants presented with secondary intestinal stricture (Table 5). The negative predictive value of CRP levels continually <10 mg/dL for the appearance of stricture was thus 100% in our study. This absence of an increase in CRP was significantly associated with the absence of secondary stricture (p<0.01). CRP levels measured initially, i.e. during the first few hours of the disease, were not significantly different between groups with and without stricture (mean 23.5 vs. 12.4 mg/L; p = 0.15). In contrast, the mean maximum CRP concentration was significantly higher in infants who developed stricture (p<0.001), as was the mean duration of the elevation of CRP levels (p<0.001; Table 5). Since surgical intervention can increase CRP serum concentration and since strictures were more frequent in cases of NEC with early surgical treatment, we also compared patients with and without stricture solely within the group of patients medically-treated during the acute phase. The duration of elevation of CRP levels was still significantly associated with the appearance of intestinal stricture in this group (p<0.01). The mean maximum CRP concentration was also higher in patients with stricture, but this difference was at the limit of significance (p = 0.05).

**Table 5:** CRP and comparison between groups with and without stricture.

	Stricture +	Stricture −	p-value
**Total population** (n)	27	21	
CRP mean maximum (mg/l)	130	58	p = 0.0007
Mean duration of CRP >10 mg/l(days)	16.7	5.9	p<0.0001
CRP <10 mg/l (n)	0	8	p = 0.01
**NEC treated medically** (n)	18	20	
CRP mean maximum (mg/l)	97	58	p = 0.05
Mean duration of CRP >10 mg/l(days)	12	5	p = 0.009
CRP <10 mg/l (n)	0	8	p = 0.036

## Discussion

This study reports a high rate of post-NEC intestinal stricture (57% of survivors), which can be explained by the very high rate of stricture in NEC cases treated surgically (91%). Nevertheless, the frequency of stricture observed after the medical treatment of NEC (47%) was also higher than that in the recent literature. The reported frequency of secondary stricture is 15–40% in survivors, 15–30% in patients treated medically and 20–43% in patients treated surgically [2], [11]–[13], [17], [20], [21], [24]–[26]. A single study reported results similar to ours in 1996, with the development of stricture in 54% of patients treated medically during the acute phase [19].

What is the reason for this high frequency in our study? The population that we studied is comparable to those in the different studies published previously, both in terms of general characteristics and in terms of the severity of NEC. The rate of surgery and the mortality rate are both similar to those reported in the principal multicenter studies (28–50% for surgery and 15–30% for mortality, up to 50% in case of surgical treatment) [1], [2], [4], [5], [7], [26]–[28]. The type of treatment during the acute phase of NEC could also have on the occurrence of post-NEC stricture. As far as surgical treatment is concerned, the techniques used are numerous and vary from center to center [29], [30]. Nevertheless, the superiority of any one technique over others has not been shown. Several studies, including two randomized multicenter trials comparing peritoneal drainage and laparotomy, have not demonstrated any significant difference between the techniques, either with respect to long-term outcome [31], [32], or with respect to the development of intestinal stricture [33], [34]. In case of laparotomy, some authors propose the resection of necrotic zones, most often with the creation of a proximal stoma [34]. Resection during the acute phase, limited to completely necrotic zones, could allow the digestive tract to be better conserved since the disappearance of necrotic lesions would lead to a decrease in the inflammatory and infectious phenomena that promote the spreading of lesions [35]. In our center, the surgical treatment of choice in all cases was laparotomy with proximal diversion without the resection of lesions. The aim of this technique was to reduce the extent of resection, based on the principle that it is difficult to predict the recovery potential of lesions observed in the acute phase. This technique is thought to increase the final length of the digestive tract conserved [36]. According to Luzzatto, among 23 cases of severe NEC requiring laparotomy with proximal diversion without resection, 21% of infants in the acute phase showed pan-intestinal damage while 36% showed pan-colitic damage. This conservative treatment permitted the extent of digestive resection to be limited to 14% [37]. In another recent report, Hunter et al. also showed that proximal diversion alone could be an option to limit bowel resection without increasing morbidity or mortality [38]. Surgery without initial resection during the acute phase leads to the secondary resection of necrotic zones that undergo stricture at a later time. This explains the high rate of secondary intestinal stricture in our study (90%) in the group of NEC cases treated surgically during the acute phase. This was accompanied by a rate of extended digestive resection comparable to that reported in the literature. However, randomized prospective study comparing this approach to other surgical option is needed to confirm the impact of this specific therapeutic strategy.

The rate of stricture in the absence of surgery during the acute phase was also high in our population (47%). The medical treatment of NEC during the acute phase is usually symptomatic, and differs little between centers, apart from the time before refeeding. When this waiting time is defined, it generally varies between 10 and 15 days and, rarely, extends up to 21 days [26], [39]–[42]. In our study, the mean duration of the cessation of feeding was 21 days. The early reestablishment of minimal enteral feeding is based on the beneficial effects of nutriments on the growth and protection of the digestive mucosa, thus promoting its repair. While the good tolerance of very early refeeding has been demonstrated in one study, it does not describe the effects of such refeeding on secondary intestinal stricture in the population studied [43]. The early reestablishment of feeding following intestinal stricture could lead to the occurrence of intestinal obstruction and septicemia due to bacterial translocation across the mucosal barrier. In our study, 7 infants were refed despite the presence of a known stricture; of these 6 became symptomatic, including 1 who developed septicemia. Similarly, Schwartz et al. describe 6 cases of stricture diagnosed by systematic contrast study but left surgically untreated. After their discharge (between 17 and 33 days), 3 infants developed a clinical picture evocative of acute digestive occlusion and only 3 remained asymptomatic [18].

In our study, all patients systematically underwent contrast study, explaining the high incidence of strictures observed, since this technique also reveals asymptomatic strictures. According to Born et al., the frequency of strictures increases from 17 to 36% when there is a systematic search for strictures before the occurrence of symptoms [43]. This systematic contrast study is not supported by all. It was proposed in the ‘80 s because of the high incidence of this complication of NEC. For certain authors, the early diagnosis of strictures by contrast study allows the mortality and morbidity (septicemia, perforation, severe intestinal obstruction) associated with a delayed diagnosis of stricture to be avoided [11], [13], [15], [17]. For others, however, it increases the number of strictures diagnosed, but does not modify their treatment: in the study by Born et al., the systematic use of contrast study doubled the number of strictures detected, but the number of strictures requiring surgery remained the same [44]. This is all the more true since the contrast study is performed at an early time point. From histological observations, it appears that the earlier the contrast study, the greater the chance that it will reveal strictures, since it detects inflammatory lesions that are likely to degenerate spontaneously [11]. Kosloske et al. recommend contrast study at 6 weeks, followed by careful refeeding [11].

One of the aims of this study was to look for risk factors for post-NEC stricture. Their identification would permit NEC cases to be subdivided into two populations: one at low risk of stricture in which early refeeding could be introduced while the contrast study could be delayed up to the 6^th^ week after NEC, and another at high risk in which it would be advisable to perform contrast study before the reestablishment of feeding. In our study, the presence of parietal signs and of thrombopenia (<100 000 platelets/mm^3^) were significantly correlated with the occurrence of stricture. However, it was above all the mean duration of systemic inflammation (defined as CRP serum concentration >10 mg/L), and conversely the absence of any elevation of CRP, that appeared to be correlated with the occurrence or the absence of post-NEC stricture, respectively. Similarly, Evrard et al. have attempted to define a scoring system for the risk of stricture following NEC [45]. In a population of 40 infants with confirmed NEC, they established a score based on clinical, biological and radiological evaluation on the 8th day of life, including 6 items: general state, ileus, abdominal erythema, duration of elevation of CRP levels, and extent and duration of pneumatosis. The maximum score was 10, and the threshold for risk was 7. Twenty infants had a score <7 (negative) and 20 had a score ≥7 (positive). They observed a single false negative and 4 false positives for the occurrence of stricture. Excluding NEC cases that were subjected to initial surgical treatment (of which all had a score ≥7 and 90% developed intestinal stricture), this score remained significantly discriminant for the prediction of stricture, as revealed by digestive contrast study [45]. One of the items in this scoring system was the elevation of CRP levels for longer than 7 days after the diagnosis of NEC. Our results confirm the interest of the kinetics of CRP elevation as a predictive factor for the occurrence of post-NEC stricture. CRP is a marker for inflammation, which plays a major role in the pathophysiology of NEC. In 1998, Pourcyrous et al. carried out a prospective study in order to determine the value of CRP in the diagnosis of NEC, in association with standard radiological examinations. In stage II NEC, CRP levels that remained elevated for more than 9 days were associated with the development of stricture or secondary sepsis, such as peritonitis or intra-abdominal abscess. Conversely, certain patients displayed digestive symptoms and parietal pneumatosis without an elevation in CRP levels: the authors observed that they showed a very rapid positive evolution with short-term antibiotic treatment and the early reestablishment of feeding [46]. In our study, 8 infants with parietal pneumatosis were negative for elevated CRP concentration even during the acute phase. They displayed a good prognosis with medium-term antibiotic therapy that was shorter than for other patients (5 days) and an earlier reestablishment of feeding (14 days). None of them developed stricture, and the discontinuation of parenteral nutrition was quicker (25 days from the beginning of NEC). Thus, our study adds some interesting data that refine the reliability of this prognosis marker, specifically to predict the development of secondary stricture. In our cohort, infants with NEC are notably more severe compared to those previously reported that could be an explanation for the high rate of strictures observed. In this very severe cohort of infants, we found that CRP has a negative predictive value of 100% when remaining below 10 mg/L in predicting subsequent strictures. The absence of an elevation in CRP levels in NEC, significantly correlated with the absence of intestinal stricture, could thus be used to determine a subpopulation for which systematic contrast study could be unnecessary. The prognostic value of other acute phase markers (procalcitonin, interleukin 6) could be tested in the future.

## Conclusion

This study demonstrates the increased frequency of intestinal stricture following NEC in case of systematic screening by digestive contrast study. This retrospective and monocentric study demonstrates the correlation between the intensity of the inflammatory syndrome and the risk of secondary intestinal stricture. It recapitulates the diagnostic and therapeutic problems posed by these intestinal strictures and highlights the importance of inflammatory process in the evolution of intestinal lesions as well the role of biological markers such as CRP in the overall prognosis of NEC.
